# STAT3 Regulates miR-384 Transcription During Th17 Polarization

**DOI:** 10.3389/fcell.2019.00253

**Published:** 2019-11-01

**Authors:** Jingjing Han, Yaping Liu, Fei Zhen, Wen Yuan, Wei Zhang, Xiaotao Song, Fuxing Dong, Ruiqin Yao, Xuebin Qu

**Affiliations:** ^1^Department of Cell Biology and Neurobiology, Xuzhou Key Laboratory of Neurobiology, Xuzhou Medical University, Xuzhou, China; ^2^Department of Neurology, the Affiliated Hospital of Xuzhou Medical University, Xuzhou, China; ^3^National Demonstration Center for Experimental Basic Medical Sciences Education, Xuzhou Medical University, Xuzhou, China

**Keywords:** miR-384, STAT3, promoter, CpG island, T helper cell 17

## Abstract

MicroRNAs are powerful regulators of gene expression in physiological and pathological conditions. We previously showed that the dysregulation of miR-384 resulted in a T helper cell 17 (Th17) imbalance and contributed to the pathogenesis of experimental autoimmune encephalomyelitis, an animal model of multiple sclerosis. In this study, we evaluated the molecular mechanisms underlying the abnormal increase in miR-384. We did not detect typical CpG islands in the *Mir384* promoter. Based on a bioinformatics analysis of the promoter, we identified three conserved transcription factor binding regions (R_I_, R_II_, and R_III_), two of which (R_II_ and R_III_) were *cis*-regulatory elements. Furthermore, we showed that signal transducer and activator of transcription 3 (STAT3) bound to specific sites in R_II_ and R_III_ based on chromatin immunoprecipitation, electrophoretic mobility shift assays, and site-specific mutagenesis. During Th17 polarization *in vitro*, STAT3 activation up-regulated miR-384, while a STAT3 phosphorylation inhibitor decreased miR-384 levels and reduced the percentage of IL-17^+^ cells, IL-17 secretion, and expression of the Th17 lineage marker Rorγt. Moreover, the simultaneous inhibition of STAT3 and miR-384 could further block Th17 polarization. These results indicate that STAT3, rather than DNA methylation, contributes to the regulation of miR-384 during Th17 polarization.

## Introduction

MicroRNAs (miRNAs) are a class of important small non-coding RNAs that either inhibit the translation of or trigger the degradation of target mRNAs by binding to 3′-untranslated regions (Saliminejad et al., 2019). About 1000 human miRNAs have been identified. They are thought to regulate more than 50% of protein-coding genes in the genome and thereby contribute to a wide array of complex cellular processes, including cell proliferation, differentiation, invasion, metastasis, apoptosis, and cell–cell communication (Selbach et al., 2008; Bayraktar et al., 2017). Furthermore, miRNAs are involved in many diseases, such as cancer, cardiovascular diseases, metabolic diseases, neurodegenerative diseases, and autoimmune diseases (Paul et al., 2018). They are used as diagnostic, prognostic, and predictive biomarkers (Jamali et al., 2018; Petrovic and Ergun, 2018; Salehi and Sharifi, 2018), and miRNA-based therapies have shown promising results in clinical trials for cancer and viral infections (Beg et al., 2017; van der Ree et al., 2017). However, the upstream mechanisms underlying the regulation of miRNAs are not well understood. In other words, the factors contributing to the disruption of specific miRNAs in certain diseases are unclear.

Most miRNA genes are transcribed by RNA polymerase II as initial stem-loop structured primary miRNAs, cleaved into precursor miRNAs by Drosha (Garcia-Lopez et al., 2013), and processed into small double-stranded RNAs by Dicer (Ha and Kim, 2014). When the miRNA-induced silencing complex is assembled, mature miRNAs are guided to destabilize mRNA and repress translation (Bartel, 2004; Bagga et al., 2005; Krol et al., 2010). Although Drosha and Dicer are indispensable during miRNA biogenesis, they cannot precisely regulate specific miRNAs in a tissue- and developmental stage-specific manner.

Tissue- or developmental stage-specific miRNAs are often associated with diseases related to specific cells and tissues. Transcription profiling of miRNAs in human tissue biopsies of different organs has shown that approximately 17% of miRNAs and miRNA families are predominantly expressed in certain tissues (Ludwig et al., 2016). Emerging evidence now indicates that epigenetic and transcriptional regulation play major roles in controlling the spatial and temporal transcription of miRNAs. In cancer, the altered transcription of many miRNAs is caused in large part by changes in DNA CpG island methylation; approximately 50% of miRNA genes are associated with CpG islands (Wang et al., 2013). For example, DNA methyltransferase (DNMT) inhibitors restore miR-127 transcription (Saito et al., 2006) and the genetic disruption of DNMTs restores the transcription of miR-124a (Lujambio et al., 2007), suggesting that DNA methylation is a major factor in miRNA transcriptional silencing in cancers.

In addition to DNA methylation, transcription factors (TFs) are thought to regulate miRNA genes in a manner similar to the regulation of protein-coding genes (Ozsolak et al., 2008). This is supported by the observation that conventional TF binding sites are located in or near promoter regions lying upstream of many miRNA genes (Turner and Slack, 2009), indicating that the promoters of specific miRNA genes can be positively or negatively controlled by TFs for activation or silencing in a tissue- or developmental stage-specific manner (Krol et al., 2010). For example, Myc activates miR-17-92 and miR-9 but inhibits the transcription of miR-15a during the proliferation and apoptosis of cancer cells (Chang et al., 2008). P53 promotes the transcription of miR-34 and miR-107 (He et al., 2007), while signal transducer and activator of transcription 3 (STAT3) down-regulates miR-520d-5p (Li et al., 2017). During nervous system development, REST is closely related to miR-124 transcription (Conaco et al., 2006).

Recent studies have shown that miR-384 is closely associated with cancer cell proliferation, metastasis, and progression (Zheng et al., 2016; Wang Y. et al., 2018; Wang Y. X. et al., 2018; Yao et al., 2019). We have previously shown that miR-384 levels are abnormally increased in the pathogenesis of experimental autoimmune encephalomyelitis (EAE) (Qu et al., 2017), a central nervous system autoimmune disease caused by inappropriate inflammation and the infiltration of IL-17-producing CD4^+^ T helper (Th17) cells (Zhu et al., 2017; Zhang et al., 2019). Enforced constitutive expression of miR-384 in CD4^+^ naïve T cells promotes polarization to the Th17 lineage, leading to severe EAE, by targeting suppressor of cytokine signaling 3 (SOCS3) (Qu et al., 2017). This previous work has clearly established that miR-384 regulates Th17 development and thereby contributes to the pathogenesis of EAE. In this study, we evaluated the upstream molecular mechanisms underlying the regulation of miR-384 and found that miR-384 could be activated by p-STAT3, thus explaining the abnormal increase in this miRNA during Th17 polarization.

## Materials and Methods

### Mice

C57BL/6 wild-type (WT) mice were purchased from SLAC Laboratory Animal Co., Ltd. (Shanghai, China), and housed under specific-pathogen-free conditions in the Xuzhou Medical University animal facility (Xuzhou, China). All experiments were performed in accordance with the Provisions and General Recommendations of the Chinese Experimental Animal Administration Legislation, as well as institutional approval from the Xuzhou Medical University Experimental Animal Ethics Committee.

### Cell Isolation, Culture, and Induction

Splenocytes (SPs), isolated from 5- to 6-week-old C57BL/6 mice, were prepared for a single-cell suspension with red blood cells depletion by ACK lysis (Beyotime, Shanghai, China). CD4^+^ T cells were isolated according to manufacturer’s instructions (Miltenyi Biotec, Bergisch Gladbach, Germany). As our previous description (Qu et al., 2016), purification of CD4^+^ naïve T cells was achieved by depletion of magnetically labeled non-naïve CD4^+^ T cells and CD44^+^ memory T cells following kit instructions (Miltenyi Biotec, Bergisch Gladbach, Germany).

For Th17 polarization, purified CD4^+^ naïve T cells were cultured for 3 days in RPMI-1640 containing 10% FBS, 1 mM glutamine, 0.1 mM β-mercaptoethanol, 1% non-essential amino acids (Sigma-Aldrich, MO, United States), anti-CD3 plus anti-CD28-coated beads (Invitrogen, CA, United States), 5 ng/ml IL-2 (R&D Systems Inc., MN, United States), 20 ng/ml IL-6, 5 ng/ml transforming growth factor-β, 10 ng/ml IL-23, 2 μg/ml anti-IL-4, and 2 μg/ml anti-interferon-γ (BD Bioscience, CA, United States). In some experiments, 10 μM AG490 (MCE, NJ, United States), 200 nM SignalSilence^®^ Stat3 siRNA II (CST, MA, United States) or miR-384 inhibitor (acauugccuaggaauuguuuaca) (Qu et al., 2017) was used.

### Flow Cytometric Analyses

Cells were incubated with Cell Stimulation Cocktail (eBioscience, CA, United States) for 5 h, then surface-stained with anti-CD4 antibody (Clone GK1.5, Miltenyi Biotec, Germany), followed by fix and permeabilization using a Fixation/Permeabilization Kit (BD Biosciences, United States). Subsequently, the cells were washed and stained with anti-IL-17 antibody (Clone TC11-18H10, Miltenyi Biotec, Germany). Tests were proceeded on MACSQuant^TM^ Flow Cytometers (Miltenyi Biotec, Germany) and analyzed with FlowJo software.

### ELISA

Quantikine ELISA kit to measure IL-17 concentration was obtained from Westang Biological Technology Co., Ltd (Shanghai, China) and used according to the manufacturer’s instructions. All samples were measured in duplicate for five times.

### Quantitative RT-PCR

After RNA were extracted using TRIzol (Invitrogen, United States) according to usual protocol, 1 μg total RNA was reverse-transcribed using a Quantscript RT kit (TIANGEN, Beijing, China) and examined with a SYBR Green real-time PCR kit (Roche, Basel, Switzerland) in lightCycler^®^ 480II System (Roche, Switzerland). The primers used were as follows: Rorγt, 5′-TGCAAGACTCATCGACAAGG and 5′-AGGGGATTCAACATCAGTGC; SOCS3, 5′-ATGGTCACCC ACAGCAAGTTT and 5′-TCCAGTAGAATCCGCTCTCCT; β-actin,5′-GAGACCTTCAACACCCCAGCC and 5′-AATGTCA CGCACGATTTCCC. Analyses of miR-384 levels were performed using SYBR Green miRNA assays (Genechem, Shanghai, China) with U6 small RNA as an internal reference for normalization. Relative expression was evaluated using 2^–ΔΔ^ ^Ct^ calculation.

### Western Blot

Cells were ultrasonically homogenized in RIPA buffer, and quantified using bicinchoninic acid protein assay kit (Beyotime, Shanghai, China). Protein samples were electrophoresed in an SDS denaturing 10% polyacrylamide gels and transferred to nitrocellulose membranes. Membranes were blocked in 0.01% PBS containing 5% BSA, incubated overnight at 4°C with anti-p-STAT3 (Clone EP2147Y), anti-STAT3 (Clone EPR787Y) and anti-GAPDH (Clone 6C5) primary antibodies (Abcam, Cambridge, United Kingdom), and then incubated in IRDye-conjugated secondary antibodies (LI-COR, CA, United States). Bands were scanned using an Odyssey Infrared Imaging System Scanner (LI-COR, United States) and images were analyzed using ImageJ software.

### Dual-Luciferase Reporter Assay

Synthesized DNA sequences (deletion constructs or binding site-mutated fragments) were cloned into the pGL4.20[luc2Puro] vector (Promega, Madison, WI, United States). The recombinant plasmids together with internal control PRL-TK Renilla vector were transfected into Jurkat cells using Lipofectamine 2000 reagent (Invitrogen, United States) following the instructions. Cells were harvested at 48 h post transfection and assayed for luciferase activity using the Dual-Luciferase Reporter Assay System (Promega, United States).

### Chromatin Immunoprecipitation Assay

ChIP assays were performed using an EZ-Magna ChIP^TM^ HiSens kit (Millipore, MA, United States) according to the manufacturer’s instructions. Chromatin was cross-linked with 1% formaldehyde for 10 min, followed by neutralization with glycine for 5 min at room temperature. Cells were then harvested, lysed, and sonicated 15 times for 4.5 s each with 9 s intervals on ice water using a Scientz-IID (Scientz, Zhejiang, China). An equal amount of chromatin was immunoprecipitated at 4°C overnight with 2 μg of p-STAT3 (Clone EP2147Y) or isotype IgG antibodies (Clone EPR25A, Abcam, United Kingdom) together with Magna ChIP protein A Magnetic Beads. Immunoprecipitated products were collected on the magnetic separator, eluted in ChIP elution buffer, and purified to obtain DNA for PCR test. Primers for PCR were listed as follows: Site 1, 5′-ATGCTATAACCACCACCA and 5′-CTTGGGATATTGTTCTGTAA; Site 2, 5′-TGCTGCCTTC TGCTTTGA and 5′-CAGGCATTGTGAACAATTTCTA; Site 3, 5′-CACTCATAAACTGGCTCG and 5′-ACTGTCTGAAGC AGTCCC.

### Electrophoretic Mobility Shift Assay

Cellular nuclear protein was extracted with Nucleoprotein Extraction Kit (Beyotime, China). A total of 12 μg of nuclear protein was added to 0.1 μM Biotin-labeled double-stranded oligonucleotides (Sangon, Shanghai, China) in 1 × EMSA/Gel-Shift binding buffer. In some trials, extra 5 μM unlabeled competitor oligonucleotide or 2 μg of anti-p-STAT3 antibody was used. Mixtures were incubated at 24°C for 20 min, analyzed by electrophoresis in 4% polyacrylamide gels at 10 V/cm, and then transferred to a nylon membrane. Membranes were UV-light cross-linked, incubated with Streptavidin-conjugated HRP, and proceeded with chemiluminescence. The probe sequences were listed as follows: Site 1, 5′-TGACCCCAGGAACTTGTATA***TGCTAGGCAA***GTACTCTATT ACAGAACAAT; Site 2, 5′-TGTATAATGTTGGTAAGTCA***TTC CTAGAAA***TTGTTCACAATGCCTGTAAC. The sequences in bold and italic show the predicted binding sites of STAT3. Site 1 mutation, 5′-TGACCCCAGGAACTTGTATA CCGACTCTTCGTACTCTATTACAGAACAAT; Site 2 mutation, 5′-TGTATAATGTTGGTAAGTCACCGACTCTTCTTGTTCAC AATGCCTGTAAC. The underline marked sequences show the mutated binding sites of STAT3.

### Bioinformatics Analysis Websites

MiR-384 and gene promoter sequences were obtained from Mirbase^1^ and UCSC^2^. CpG islands prediction was analyzed by EMBOSS Cpgplot^3^, MethPrimer^4^, and Sequence Manipulation Suite^5^. Transcription factor binding sites were predicted using the ECR Browser^6^ and JASPAR database^7^.

### Statistical Analyses

The results were expressed as mean ± standard deviation (SD) and analyzed by SPSS 17.0. Independent sample *t* tests were used to evaluate differences between groups. Two-way analysis of variance followed by Bonferroni’s *post hoc* test was used for multiple comparisons. A *P* value of 0.05 or less was considered significant.

## Results

### CpG Islands Are Absent in the *Mir384* Promoter

**FIGURE 1:**
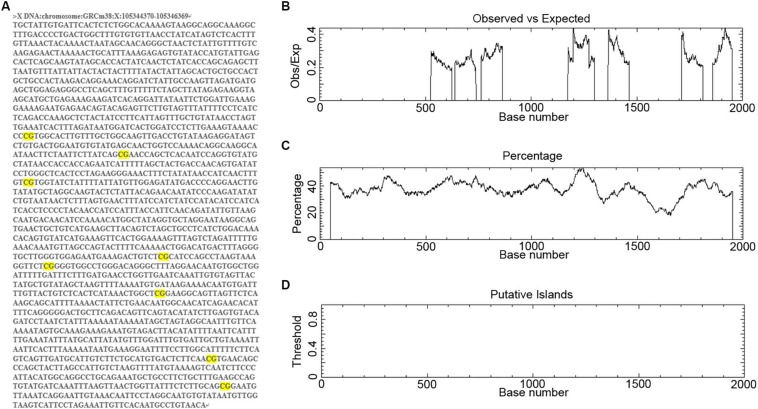
Analysis of CpG islands in *Mir384* promoter. **(A)** The DNA sequence 2000 bp upstream of *Mir384* in chromosome X. CG dinucleotides are marked in bright yellow. **(B–D)** *Mir384* promoter sequence is analyzed by EMBOSS Cpgplot for CpG islands prediction, and the ratio of observed to expected **(B)**, percentage of CG **(C)**, and putative CpG island **(D)** are shown.

To determine the regulatory mechanisms controlling miR-384 transcription, we obtained the DNA sequence 2000 bp upstream of *Mir384* (Figure 1A). We scanned this sequence for promoter methylation, a major mechanism underlying miRNA activation or silencing. We found eight groups of CpG dinucleotides separated from each other (Figure 1A). Further analysis using EMBOSS Cpgplot showed that in every 100-nucleotide window, the ratio of observed to expected (Obs/Exp) CpG sites was less than 0.45 (Figure 1B) and the percentage of CpG sites was less than 55% (Figure 1C). Thus, no putative CpG island was identified in this sequence (Figure 1D) according to established criteria (Island size >100 bp, GC percentage >50, and Obs/Exp >0.6). Similarly, no CpG islands were found in this sequence based on analyses using MethPrimer and Sequence Manipulation Suite (data not shown). Based on these results, we hypothesized that miR-384 transcription was not regulated by promoter methylation.

### STAT3 Binds Directly to Specific Sites in the *Mir384* Promoter

**FIGURE 2:**
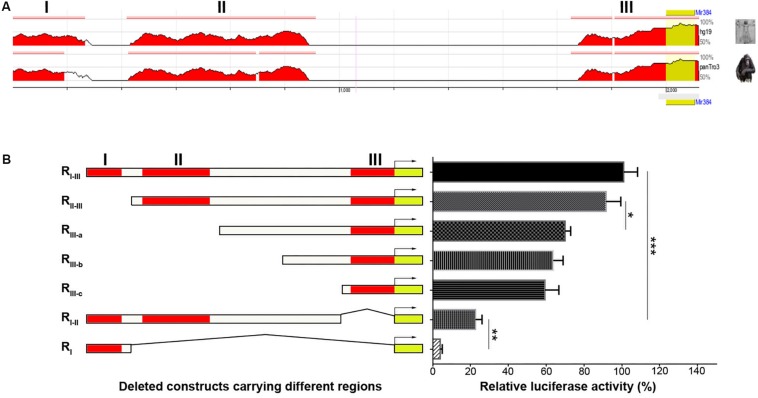
Analysis of TFs binding in different regions of *Mir384* promoter. **(A)** The homologous binding sites of TFs in *Mir384* promoter among the species of mouse, human, and chimp. This sequence is divided into three regions (I, II, and III) according to the binding sites. Peaks represent the level of homology. **(B)** Effects of different binding region deletion on transcriptional activity by luciferase assay. On the left side is a schematic representation of the deleted DNA sequences carrying different regions. The right panel shows luciferase activity normalized to Renilla luciferase activity. Data are presented as mean ± standard deviation. ^∗^*P* < 0.05. ^∗∗^*P* < 0.01. ^∗∗∗^*P* < 0.001. Data are representative of three experiments done in triplicate.

The lack of CpG islands in the *Mir384* promoter suggests that miR-384 might be regulated by TFs. Using ECR Browser, we identified three predicted TF binding site regions (I, II, and III) in this promoter sequence with high conservation across three closely related mammalian taxa: mice, humans, and chimps (Figure 2A). Next, we constructed DNA fragments carrying variant regions (Figure 2B, left) for luciferase assays and found that the deletion of region I had no obvious effect on transcriptional activity, while a lack of region II attenuated transcription and the construct lacking region III exhibited only about one-fifth of the total activity observed for the construct carrying all three regions (Figure 2B). Moreover, the simultaneous deletion of region II and III resulted in highly decreased transcriptional activity (Figure 2B). These data suggest that region II and, to a greater extent, region III, have important roles in transcription.

**FIGURE 3:**
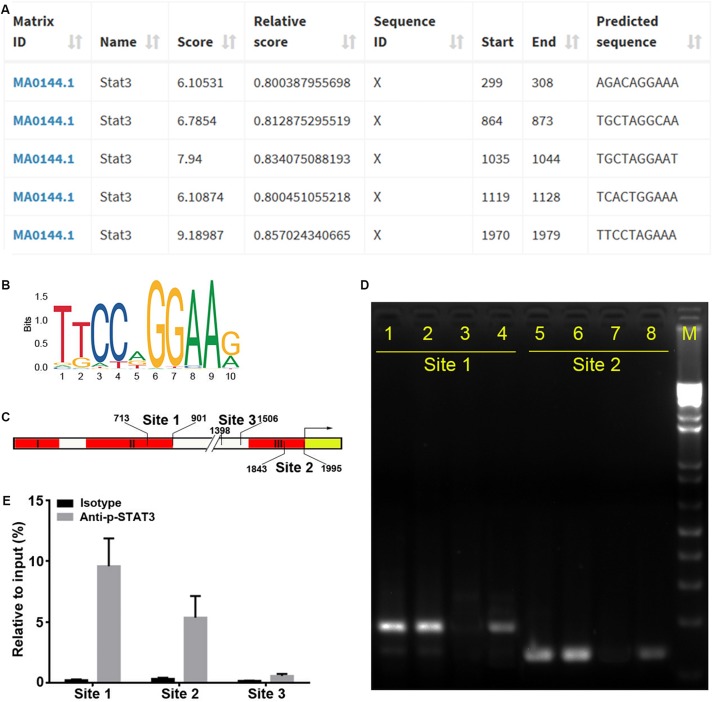
STAT3 binds to *Mir384* promoter. **(A)** Analysis of STAT3 binding sites in the *Mir384* promoter by JASPAR database. **(B)** Sequence logo of STAT3 binding sites. **(C)** Pattern diagram shows the locations of site 1, site 2, site 3, and primers for ChIP-PCR. **(D)** Gel electropherogram of ChIP-PCR products at site 1 and site 2. Lanes 1 and 5, immunoprecipitation by anti-RNA Polymerase II antibody. Lanes 2 and 6, input. Lanes 3 and 7, immunoprecipitation by isotype IgG. Lanes 4 and 8, immunoprecipitation by anti-p-STAT3 antibody. M, DNA marker. **(E)** ChIP-qRT-PCR assay to analyze p-STAT3 binding to the *Mir384* promoter at three sites. DNA isolated from immune-precipitated materials is amplified using qRT-PCR. The values are normalized to the input for each sample.

To identify the precise TFs that bind to regions II and III to regulate miR-384 transcription, we analyzed the *Mir384* promoter sequence using the JASPAR database and identified five STAT3 binding motifs (Figures 3A,B), one (864 to 873, site 1) located in region II and another (1970 to 1979, site 2) located in region III (Figure 3C). A ChIP analysis showed that the site 1 and site 2 fragments were significantly enriched, while the site 3 fragment with no STAT3 binding motif was underrepresented after p-STAT3 immunoprecipitation (Figures 3D,E), suggesting that p-STAT3 binds to both site 1 and 2 but not to site 3.

**FIGURE 4:**
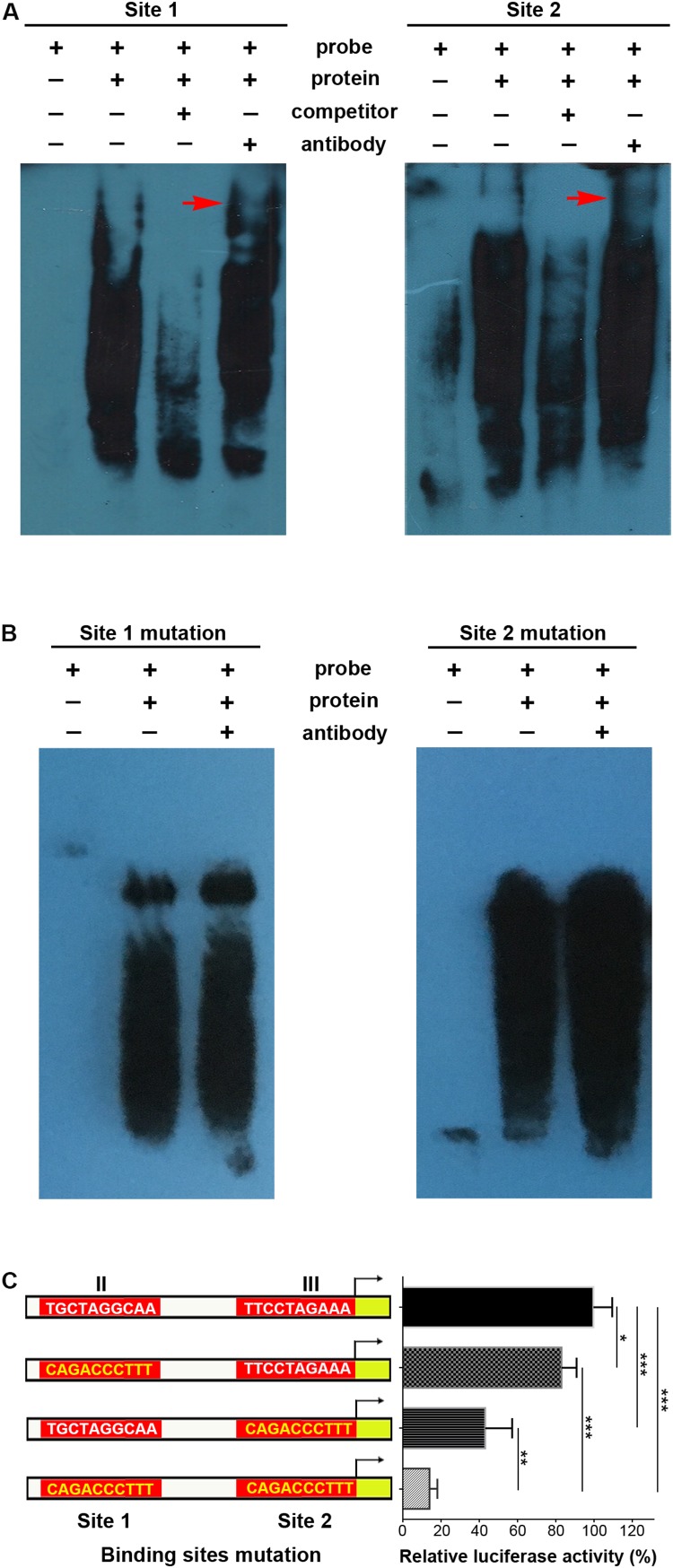
STAT3 binds with special sites in *Mir384* promoter. **(A,B)** EMSA shows the interaction between p-STAT3 and specific probe sequences containing site 1, site 2, mutated site 1, or mutated site 2. Binding complexes are identified by supershifted bands (red arrows) with antibody against p-STAT3. **(C)** Effects of binding site mutagenesis on transcriptional activity by luciferase assay. On the left side is a schematic representation of the WT (white) and special site-directed mutation (yellow) constructs at site 1 in region II and site 2 in region III. The right panel shows the luciferase activity normalized to Renilla luciferase activity. Data are presented as mean ± standard deviation. ^∗^*P* < 0.05. ^∗∗^*P* < 0.01. ^∗∗∗^*P* < 0.001. Data are representative of three experiments done in triplicate.

Supershifted EMSA bands further indicated that p-STAT3 bound to both site 1 and site 2 probes, while these bands disappeared when using unlabeled competitor probes (Figure 4A). When mutations were introduced in sites 1 and 2, the supershifted bands disappeared (Figure 4B). Furthermore, site-specific mutations in either site 1 or site 2 could significantly decrease transcriptional activity, and simultaneous mutations in both sites further inhibited transcription (Figure 4C). Taken together, these data demonstrate that p-STAT3 can bind to conserved sites in *cis*-regulatory elements of the *Mir384* promoter.

### STAT3 Regulates miR-384 Transcription During Th17 Polarization

**FIGURE 5:**
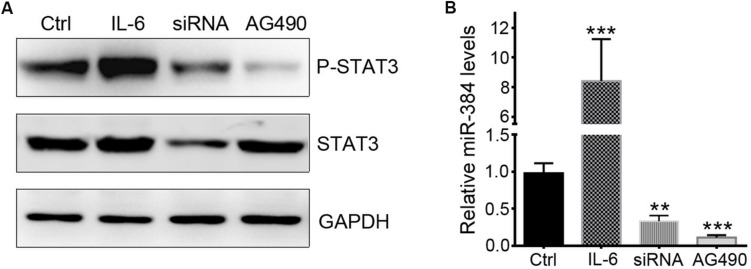
STAT3 regulates miR-384 transcription. CD4^+^ naïve T cells are cultured with IL-6, siRNA, or AG490, and then p-STAT3 and STAT3 levels are tested by Western blot **(A)**, and miR-384 transcription is measured by qRT-PCR **(B)**. Data are presented as mean ± standard deviation. ^∗∗^*P* < 0.01. ^∗∗∗^*P* < 0.001. Data are representative of three experiments done in triplicate.

To determine whether STAT3 activation could regulate miR-384 transcription, we used IL-6 to activate STAT3, siRNA to knockdown STAT3 expression, or AG490 to inhibit STAT3 phosphorylation with more than 95% selectively by blocking JAK2 with no effect on STAT3 mass and cell viability (Dowlati et al., 2004). In CD4^+^ naïve T cells, IL-6 could obviously promote the phosphorylation of STAT3, while siRNA and AG490 significantly decreased p-STAT3 levels (Figure 5A). A qRT-PCR analysis showed that IL-6 increased miR-384 levels by more than eightfold, while miR-384 levels reduced to one-third or one-sixth of control levels when siRNA or AG490 was used, respectively (Figure 5B), suggesting that STAT3 activation mediates miR-384 transcription.

**FIGURE 6:**
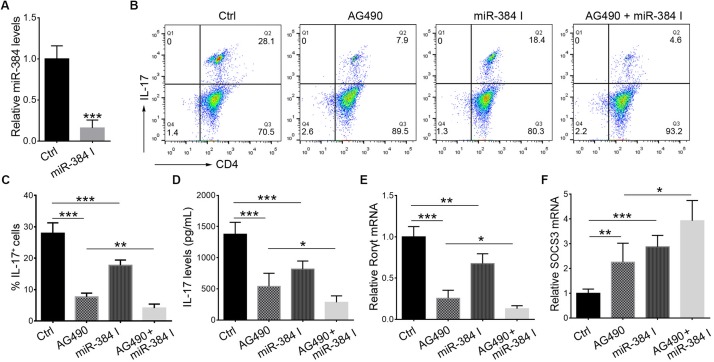
STAT3 and miR-384 mediate Th17 polarization. CD4^+^ naïve T cells are cultured in Th17 polarizing conditions with AG490 or miR-384 inhibitor (miR-384 I) for 3 days, and then miR-384 transcription is measured by qRT-PCR **(A)**, percentage of stimulated IL-17^+^ cells is tested by flow cytometry **(B,C)**, dose of IL-17 in culture supernatant is measured by ELISA **(D)**, and Rorγt mRNA **(E)** and SOCS3 mRNA **(F)** levels are analyzed by qRT-PCR. Data are presented as mean ± standard deviation. ^∗^*P* < 0.05. ^∗∗^*P* < 0.01. ^∗∗∗^*P* < 0.001. Data are representative of three experiments done in triplicate.

During Th17 polarization *in vitro*, treatment with AG490 or a miR-384 inhibitor (Figure 6A) could suppress IL-17^+^ cell cytopoiesis, with decreased IL-17 secretion and Rorγt expression but up-regulated SOCS3 (Figures 6B–F). Moreover, the simultaneous inhibition of STAT3 and miR-384 further reduced IL-17^+^ cell generation, IL-17 concentrations, and Rorγt expression but increased SOCS3 (Figures 6B–F). These data indicate that the STAT3-mediated regulation of miR-384 transcription plays a role in Th17 polarization.

## Discussion

Tissue- or developmental stage-specific alterations in miRNAs are associated with diseases. Studies of miRNAs in tumors have been fruitful, including the identification of hundreds of thousands of miRNAs related to tumorigenesis (Kulkarni et al., 2019), proliferation, migration (Li et al., 2010), apoptosis, necroptosis (Shirjang et al., 2019), and metabolism (Subramaniam et al., 2019). Recent findings have suggested that the dysregulation of miRNAs is associated with several central neurological disorders, such as Alzheimer’s disease (Angelucci et al., 2019), Parkinson’s disease (Reddy et al., 2019), and multiple sclerosis (MS) (Marangon et al., 2019). Our previous studies have shown that certain miRNAs are closely related to the development of EAE, a mouse model of MS. Genome-wide transcription profiling indicates that miR-30a levels are substantially decreased in both patients with MS and mice with EAE, but miR-384 levels are significantly increased. Enforced constitutive expression of miR-30a *in vivo* results in fewer Th17 cells and alleviates EAE via IL-21R, while the expression of miR-384 *in vivo* leads to severe EAE due to SOCS3 inhibition (Qu et al., 2016, 2017). However, these studies have not resolved the factors contributing to the abnormal levels of miR-30a and miR-384 in EAE conditions. Promoter-associated CpG island methylation is a major mechanism underlying miRNA regulation and about 50% of miRNA genes are associated with CpG islands (Wang et al., 2013). However, we did not identify CpG island regions in 2000 bp upstream of *Mir384* using bioinformatics tools, suggesting that promoter methylation status does not explain differences in transcription. Thus, neither methylation-specific PCR nor bisulfite-modified DNA sequencing were performed. We detected potential binding motifs of STAT3, a key TF regulating Th17 polarization, in *cis*-regulatory elements within the *Mir384* promoter. Owing to the high false-positive and false-negative rates for prediction algorithms, we performed an additional ChIP assay, EMSA, and binding site mutagenesis combined with luciferase reporter assays to show that p-STAT3 bound directly to the *Mir384* promoter in regions II and III. Furthermore, STAT3 activation up-regulated miR-384, while a STAT3 phosphorylation inhibitor decreased miR-384, with reductions in the percentage of IL-17^+^ cells, IL-17 secretion, and expression of the Th17 lineage marker Rorγt, suggesting that STAT3 activation directly regulates miR-384 transcription during Th17 polarization.

STAT3, a member of the STAT family of TFs, is an important constitutive signaling molecule for many key genes involved in multiple biological functions. It targets many miRNAs (Rokavec et al., 2014; Liao et al., 2015). For example, STAT3-induced miR-92a and miR-520d-5p suppression regulate cancer growth and survival (Chen et al., 2017; Li et al., 2017), and the inactivation of the phosphorylation of STAT3 decreases miR-155-5p and its anti-cancer properties (Zheng et al., 2018). Inhibition of the STAT3-associated pathway not only reduces miR-21 levels in cells but also inhibits the release of miR-21-enriched exosomes (Chuang et al., 2019). Using STAT3 cardiomyocyte-deficient mice, STAT3-mediated decreases in miR-34b and miR-337 play key roles in cardio-protection (Pedretti et al., 2019). Mechanistically, p-STAT3 binds to the promoter region of miR-199a-2 for regulation (Zhou et al., 2019). Three functional binding sites of STAT3 in the *Mir21* promoter region mediate angiogenesis (Chen et al., 2019). During Th17 polarization, IL-6-activated STAT3 has particularly important roles in the expression of Rorγt, a Th17 lineage marker. However, it is not clear whether STAT3 regulates Th17-related miRNAs. Our results showed that IL-6 stimulation resulted in the phosphorylation of STAT3 and activation of miR-384, thus potentially explaining the abnormal increase in miR-384 levels in Th17 and EAE.

**FIGURE 7:**
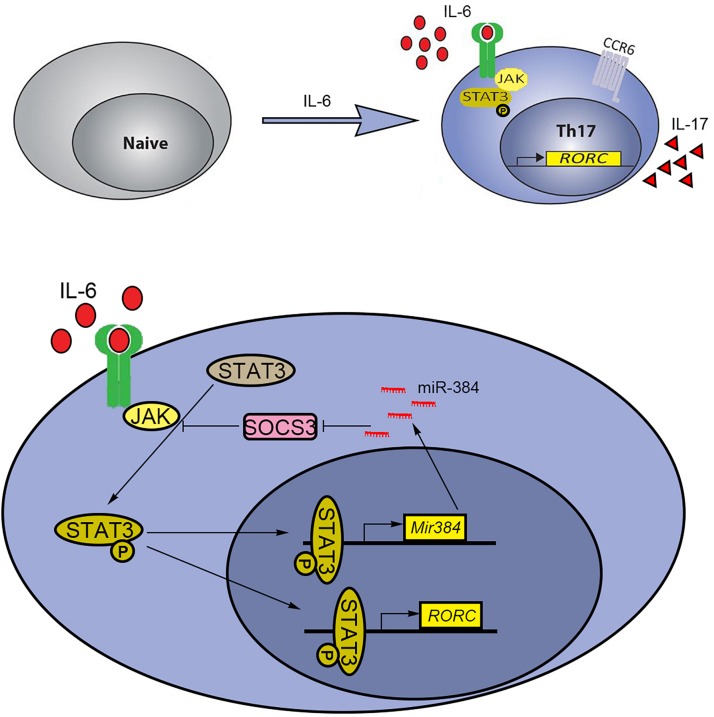
Schematic model of STAT3 and miR-384 regulating Th17 polarization. IL-6-induced activation of STAT3 promotes transcription of miR-384 and Rorγt (RORC). The increased miR-384 further activates STAT3 via targeting SOCS3, resulting in promoted Th17 polarization.

T helper cell 17 development relies on key cytokines, including IL-6, IL-21, and IL-23, via STAT3 activation for gain of effector Th17 cell functions, such as the expression of the inflammatory cytokines IL-17, IFN-γ, and GM-CSF. Mice with STAT3 knockout in Th17 cells are resistant to the development of EAE because the STAT3 deficiency decreases Th17 counts in lymph nodes and the central nervous system (Yang et al., 2007). Thus, targeting STAT3 signaling is a potential strategy to alleviate Th17-related diseases. Our previous results showed that miR-384 was an upstream factor that directly inhibited SOCS3 during Th17 polarization (Qu et al., 2017). Furthermore, miR-384 is a direct target of STAT3 and is induced by IL-6. However, the repression of Th17 polarization when miR-384 was inhibited was weaker than that observed for STAT3 inhibition, suggesting that miR-384 is not the only downstream target of STAT3, which can directly regulate Rorγt (Qu et al., 2012; Tanaka et al., 2014). Since STAT3 is constitutively expressed in cells, it is possible that miR-384 is co-regulated by other TF complexes under specific cellular contexts or chromatin features; this is supported by our analysis showing that the simultaneous inhibition of STAT3 and miR-384 further blocked Th17 polarization. Based on our findings, we propose a schematic model by which IL-6 induces the STAT3-mediated activation of miR-384 and its downstream target SOCS3 to partially regulate Th17 polarization (Figure 7).

## Data Availability Statement

All datasets generated for this study are included in the article/supplementary material.

## Ethics Statement

The animal study was reviewed and approved by Xuzhou Medical University Experimental Animal Ethics Committee.

## Author Contributions

XQ and RY designed the study, performed experiments, analyzed the data, and wrote and reviewed the manuscript. JH, YL, FZ, WY, WZ, XS, and FD performed the experiments. All authors read and approved the final manuscript.

## Conflict of Interest

The authors declare that the research was conducted in the absence of any commercial or financial relationships that could be construed as a potential conflict of interest.

## Footnotes

1
http://www.mirbase.org/

2 http://genome.ucsc.edu/ENCODE/

3 http://www.ebi.ac.uk/emboss/cpgplot/

4 http://www.urogene.org/methprimer

5 http://www.bioinformatics.org/sms2/

6 https://www.dcode.org/

7 https://jaspar.genereg.net/
